# A Study on the Interfacial Reactions between Gallium and Cu/Ni/Au(Pd) Multilayer Metallization

**DOI:** 10.3390/ma16186186

**Published:** 2023-09-13

**Authors:** Byungwoo Kim, Chang-Lae Kim, Yoonchul Sohn

**Affiliations:** 1 Department of Welding and Joining Science Engineering, Chosun University, Gwangju 61452, Republic of Korea; 2 Solder R&D Team, MK Electron Co., Ltd., Yongin 449-812, Republic of Korea; 3 Department of Mechanical Engineering, Chosun University, Gwangju 61452, Republic of Korea

**Keywords:** gallium, intermetallic compound, interfacial reaction, X-ray diffraction, scanning electron microscopy

## Abstract

This research introduces low-temperature soldering of Ga with practical metallization structures, namely, Cu/Ni/Pd and Cu/Ni/Au, applied to contemporary microelectronic packages. Through these multilayer configurations, the study investigates the stability of the Ni diffusion barrier by examining changes in the interfacial microstructure as Ni is consumed. The interfacial reactions are conducted across a temperature spectrum of 160, 200, 240, and 280 °C, with reaction durations ranging from 30 to 270 min. Valuable insights for low-temperature soldering with Ga are extracted from the data. At lower reaction temperatures, the presence of Ga-rich intermetallic compounds (IMCs), specifically Ga_x_Ni (x = 89 to 95 at%), on the Ga_7_Ni_3_ layer is notably confirmed. As the reaction temperature and duration increase, the gradual consumption of the Ni layer occurs. This gives rise to the formation of Ga-Cu IMCs, specifically CuGa_2_ and γ3-Cu_9_Ga_4_, beneath the Ga-Ni IMC layer. Concurrently, the gap between the Ga-Ni and Ga-Cu IMC layers widens, allowing molten Ga to infiltrate. The rate of Ga_7_Ni_3_ growth follows a time exponent ranging approximately from 1.1 to 1.7. This highlights the significant influence of interface reaction-controlled kinetics on Ga_7_Ni_3_ IMC growth. The activation energy for Ga_7_Ni_3_ growth is determined to be 61.5 kJ/mol. The growth of Ga_7_Ni_3_ is believed to be primarily driven by the diffusion of Ga atoms along grain boundaries, with the porous microstructure inherent in the Ga_7_Ni_3_ layer providing additional diffusion pathways.

## 1. Introduction

Liquid gallium (Ga) and Ga-based alloys, such as the eutectic Ga-In and galinstan [1,2], have captured widespread interest due to their inherent qualities of being deformable, conducive to miniaturization, amenable to low-temperature processing during fabrication, and low in toxicity. The escalating demand for pliable and wearable devices, encompassing prosthetics and implantable technologies, has ignited research efforts and precipitated a surge in the production of adaptable electronic devices [3,4]. Recently, several promising circuits [5,6,7] and electronic components [8,9,10] that incorporate liquid metals capable of being bent or stretched have been showcased. Additionally, foundational techniques for manipulating liquid metals have been explored to unlock their potential applications.

When constructing an electronic device, it is essential to establish electrical connections between the chip and substrate. Soldering, a foundational interconnection technique in microelectronic packaging, comes into play. There has been a continuous drive to develop diverse alloy combinations involving Sn-based solders like Sn-3.0Ag-0.5Cu and Sn-58Bi. These alloys aim to facilitate soldering processes at lower temperatures [11]. By achieving this, these alloys could contribute to energy conservation and a reduced risk of component damage. Given their comparatively low melting points and capability to form intermetallic compounds (IMCs) with various metals, liquid Ga and Ga-based alloys have been explored as potential soldering materials in microelectronics.

Recently, reports on the reaction studies between liquid gallium and other metals widely used in the electronics industry have been gradually increasing. Among them, many studies have concentrated on Cu, and other studies have been reported on the reaction with metals such as Ni, Pd, and Au [12,13,14,15,16,17]. In the study conducted by S.K. Lin et al. [12], an investigation was carried out involving diffusion couples of Cu/Ga and Cu/Ga/Cu. The researchers identified the presence of two IMCs, namely, Cu_9_Ga_4_ and CuGa_2_. They also determined time exponents of 1.0 at 160 °C and approximately 2.0 for the temperature range of 200–240 °C. In a research effort led by Chen et al. [13], an investigation involving diffusion couples of Cu/Ga/Ni was conducted. The study unveiled the emergence of a Ga_7_Ni_3_ phase within the temperature range of 200–350 °C, while a Ga_3_Ni_2_ phase was observed at 500 °C. The researchers noted that the consumption of Ga displayed a linear dependence on the square root of the reaction time. In another study, the interaction between Cu/Ni/Ga/Ni/Cu reaction couples was examined [14]. The outcome revealed a complete transformation of these couples into solid-solution joints, specifically Cu/face-centered cubic (fcc)-(Ni, Cu, Ga)/Cu. The process involved a reaction between Ga and Ni, leading to the predominant formation of the Ga_7_Ni_3_ phase at 300 °C. This phase formation exhibited a time exponent of approximately 1.0. Lee et al. [15] reported detailed investigations on the interfacial reactions between Ga and Ni at temperatures of 250–350 °C. The IMC double layer observed after the reactions contained a Ga_7_Ni_3_ bottom layer formed during the reactions, and a Ga_x_Ni top layer (x = 89–95 at%) precipitated during cooling. Ga_7_Ni_3_ growth occurred only in the vertical direction, without lateral coarsening and merging between the rods. The time exponents were measured at 1.1–1.5, with an activation energy of 49.1 kJ/mol.

In this study, practical under-bump metallization (UBM) structures of Cu/Ni/Pd and Cu/Ni/Au applied to modern microelectronic packages are introduced to secure practical experimental data. Using two different wetting layers, Pd and Au, we investigated whether the Ni layer could act as a sufficient diffusion barrier while reacting with Ga. In addition, by observing the change of the interfacial microstructure according to Ni consumption, useful data can be provided in the case of Ga soldering in the future.

## 2. Materials and Methods

### 2.1. Materials and Specimen Preparation

High-purity gallium metal (99.999%) and copper foil (99.999%, with a thickness of 0.5 mm) were procured from Alfa Aesar (Haverhill, MA, USA). Electrodeposition of Ni/Pd (10 μm/0.5 μm) or Ni/Au (5 μm/0.1 μm) multilayer coatings was carried out at SJ Company in Korea. For the creation of test specimens to study the interactions between gallium and the multilayer UBMs, 0.05 g of gallium metal was carefully placed on the UBMs that had been deposited on the 0.5 mm thick copper foil, with dimensions measuring 5 mm × 10 mm.

The interfacial reactions were executed within a temperature range of 160, 200, 240, and 280 °C, utilizing a convection oven, and the reactions lasted between 30 and 270 min. Following the reaction process, the specimens were allowed to cool in ambient air for a duration of 2 min, subsequently being solidified by freezing for over 30 min at a temperature of −20 °C. A comprehensive step-by-step description of the experimental procedure can be found in reference [15]. The processed specimens were then embedded in epoxy and subjected to grinding and polishing procedures, enabling the examination of their cross-sectional features. For the purpose of clearly distinguishing the IMCs from the unreacted gallium, a gentle etching process using a diluted hydrochloric acid solution (10 vol.%, mixed in deionized water) was performed.

### 2.2. Characterization

Cross-sectional micrographs of the samples were captured using scanning electron microscopy (SEM). Moreover, the IMCs were characterized through energy-dispersive X-ray (EDX) analysis. The thickness of the IMC layer observed in the micrographs was determined by employing image analysis software. The thickness of the layer was computed by dividing the total phase area by its length. Average values were obtained based on measurements taken from five distinct areas on each reaction sample. Furthermore, precise identification of the interfacial IMCs was accomplished using X-ray diffraction (XRD). For the creation of XRD specimens, the interfacial IMCs present on the surface were unveiled through complete etching of the unreacted gallium after the interfacial reactions.

## 3. Results and Discussion

### 3.1. Formation and Growth of the Interfacial IMCs

**Figure 1:**
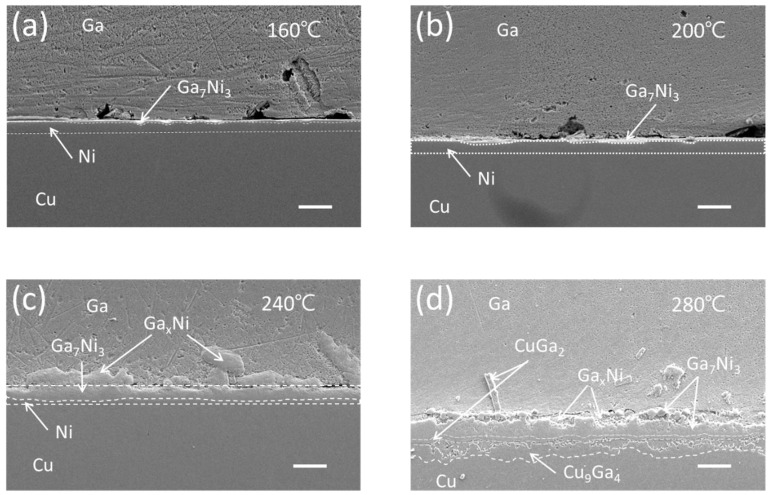
Cross-sectional SEM micrographs of the Cu/Ni/Au-Ga interfaces after 30 min reaction at reaction temperatures of (**a**) 160 °C, (**b**) 200 °C, (**c**) 240 °C, and (**d**) 280 °C (scale bar: 20 µm).

Figure 1 illustrates the interfacial microstructures following a 30-min reaction between the Cu/Ni/Au UBM and Ga at temperatures of 160, 200, 240, and 280 °C. While the Ni was not entirely consumed, two distinct types of IMCs emerged at the reaction interface: the Ga_x_Ni (x = 89~95 at%) and Ga_7_Ni_3_ phases. It was documented by Lee et al. [15] that the Ga-rich phase, Ga_x_Ni, with nonstoichiometric composition, was formed during the cooling stage and deposited onto the Ga_7_Ni_3_ layer, while the Ga_7_Ni_3_ phase developed at the reaction temperature.

At temperatures below 240 °C, the Ga_7_Ni_3_ IMC persisted as a stable layer over the residual nickel, while at 280 °C, the nickel layer underwent complete consumption. At this higher temperature, two Cu-Ga IMCs, namely CuGa_2_ and Cu_9_Ga_4_, were observed to form beneath the Ni-Ga IMCs. Previous reports have documented that the reaction between Ga and Cu at room temperature yields the CuGa_2_ intermetallic phase [18,19]. Lin et al. [12] noted the presence of a double layer comprising CuGa_2_ and γ3-Cu_9_Ga_4_ IMCs during the reaction at 160–240 °C, whereas a single γ3-Cu_9_Ga_4_ IMC was observed during the reaction at 280–300 °C. Additionally, Chen et al. [13] discovered γ3-Cu_9_Ga_4_ beneath CuGa_2_ during the reaction at 200 °C, whereas γ2-Cu_9_Ga_4_ and γ1-Cu_9_Ga_4_ were identified at 350 °C and 500 °C, respectively.

The Ga-Cu binary phase diagram encompasses four γ-brass polymorphs (γ, γ1, γ2, and γ3) [20,21]. These polymorphs can be characterized in terms of two clusters with the same fundamental configuration but a different distribution of atoms. Transformations between γ and γ1 involve order–disorder transitions, while the presence of vacancies is involved with γ2 and γ3 [21]. Here, the concentration of Ga within the γ phases progressively rises from approximately 30 at% to 40 at% or higher as it moves in the direction of γ1 to γ2 to γ3. Based on the research outcomes, it is evident that the CuGa_2_ phase emerges at room temperature, a combination of CuGa_2_ and γ3-Cu_9_Ga_4_ phases coexist at around 160–300 °C, and γ2-Cu_9_Ga_4_ and γ1-Cu_9_Ga_4_ phases manifest at high temperatures of 350 and 500 °C, respectively. In alignment with these findings, the present study also established the formation of a CuGa_2_/γ3-Cu_9_Ga_4_ IMC double layer as a result of the reaction between Ga and Cu at 280 °C for 30 min.

**Figure 2:**
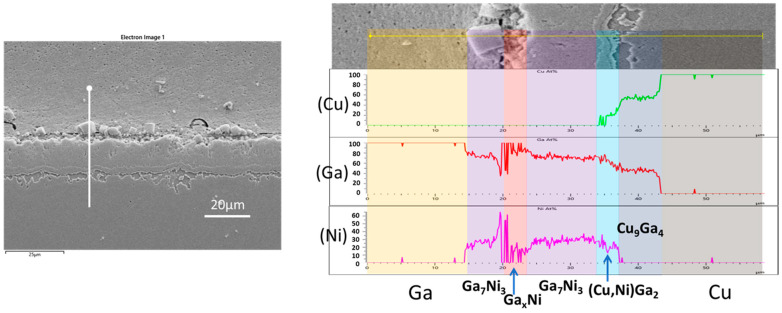
Compositional analysis using an EDX line scan of the Cu/Ni/Au-Ga specimen that reacted at 280 °C for 30 min.

To validate the IMC phases that developed at the interface of the Cu/Ni/Au-Ga specimen and reacted at 280 °C, an EDX line scan was performed in a distinct location from that shown in Figure 1d. The result of this analysis is presented in Figure 2. Here, a significant portion of the Ni had undergone transformation into a substantial Ni_7_Ga_3_ layer, on top of which a thin layer of the Ga_x_Ni IMC was observed. Some chunks of Ni_7_Ga_3_ had detached and dispersed into the bulk Ga. A distinct band of the CuGa_2_ phase, inclusive of some nickel, represented as (Cu,Ni)Ga_2_, was evident below the Ni_7_Ga_3_ layer. Additionally, an intermetallic layer of γ3-Cu_9_Ga_4_ was found positioned between the (Cu,Ni)Ga_2_ band and the Cu substrate. The presence of the Cu_9_Ga_4_ IMC with a gallium content exceeding 40 at% confirmed its classification as the γ3 phase.

**Figure 3:**
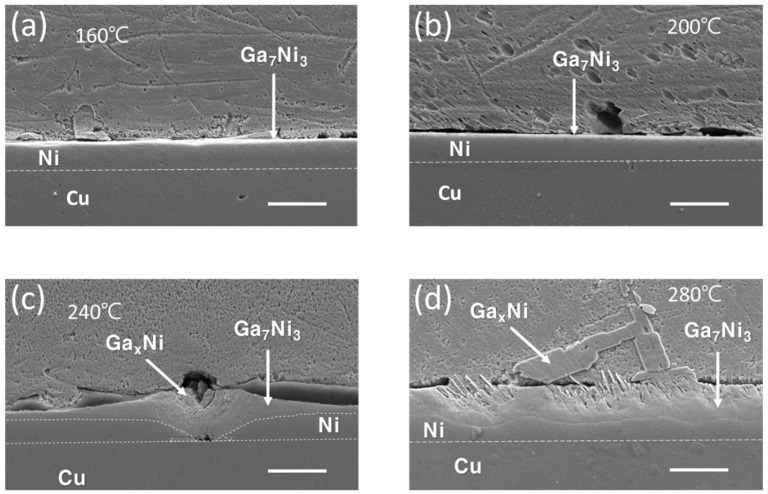
Cross-sectional SEM micrographs of the Cu/Ni/Pd-Ga interfaces after 30 min reaction at reaction temperatures of (**a**) 160 °C, (**b**) 200 °C, (**c**) 240 °C, and (**d**) 280 °C (scale bar: 20 µm).

Figure 3 presents the interfacial microstructures resulting from a 30 min reaction between the Cu/Ni/Pd UBM and Ga at temperatures of 160, 200, 240, and 280 °C. Due to the substantial thickness of the Pd wetting layer (0.5 μm) and the Ni layer (10 μm), the Ni layer was not completely consumed even after a 30 min reaction at 280 °C. Notably, the Ga_7_Ni_3_ IMC layer was prominently positioned on top of the Ni layer, while the Ga_x_Ni IMCs rested above it, with some of these positioned in the bulk Ga.

**Figure 4:**
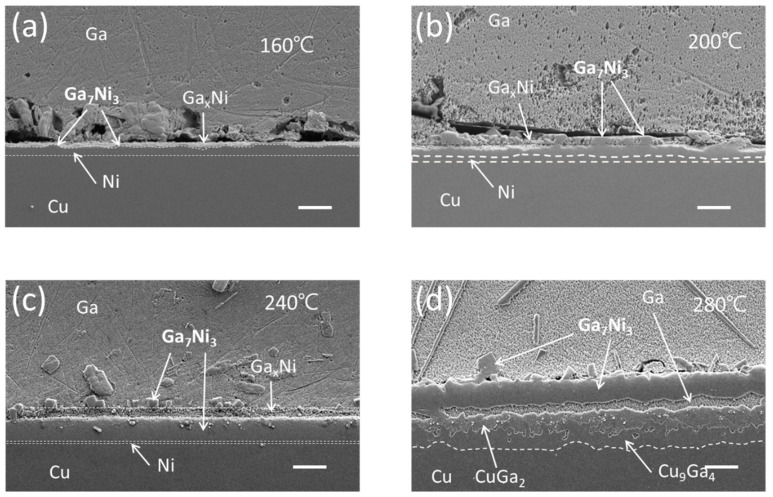
Cross-sectional SEM micrographs of the Cu/Ni/Au-Ga interfaces after 210 min reaction at reaction temperatures of (**a**) 160 °C, (**b**) 200 °C, (**c**) 240 °C, and (**d**) 280 °C (scale bar: 20 µm).

Figure 4 presents the interfacial microstructures resulting from a 210 min reaction between the Cu/Ni/Au UBM and Ga at temperatures of 160, 200, 240, and 280 °C. Given the extended reaction duration of 210 min and the substantial consumption of Ni, the residual Ni layer thickness was notably diminished in specimens exposed to temperatures below 240 °C. Among these specimens, the presence of the Ga_x_Ni layer on top of the Ga_7_Ni_3_ layer was most distinctly verified in the specimen subjected to 240 °C. For the reactions at 280 °C, by comparing the specimen that reacted for 210 min in Figure 4d with that which reacted for 30 min in Figure 1d, it becomes evident that the thickness of the Ga-Cu IMCs, CuGa_2_, and Cu_9_Ga_4_ underwent expansion. Additionally, Ga infiltration occurred as a distinct gap that emerged between this Ga-Cu IMC layer and the Ga_7_Ni_3_ IMC layer.

**Figure 5:**
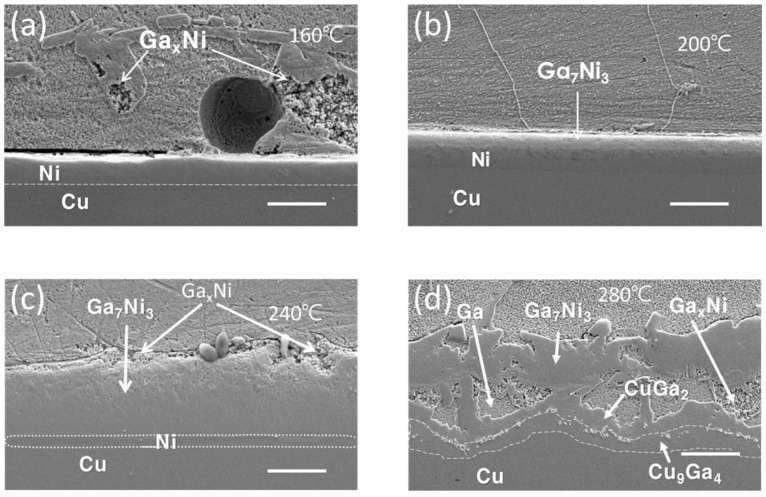
Cross-sectional SEM micrographs of the Cu/Ni/Pd-Ga interfaces after 210 min reaction at reaction temperatures of (**a**) 160 °C, (**b**) 200 °C, (**c**) 240 °C, and (**d**) 280 °C (scale bar: 20 µm).

Figure 5 depicts the interfacial microstructures that emerge after a 210 min reaction between the Cu/Ni/Pd UBM and Ga at temperatures spanning 160, 200, 240, and 280 °C. At temperatures below 240 °C, the formation of Ga_x_Ni and Ga_7_Ni_3_ IMCs showcases similarities that are not notably divergent from their counterparts in the Ni/Au UBM. In instances where the Ga_x_Ni IMC is more closely identified, two distinct shapes become evident. One takes the form of minute powdery clusters positioned on top of the Ga_7_Ni_3_ layer, as depicted in Figure 4c and Figure 5c. The other manifestation entails these clusters being embedded in a cohesive mass within the bulk Ga, as shown in Figure 3d and Figure 5a. Upon closer inspection, it is distinguished by its porous and sparsely arranged internal structure, revealing the existence of the aforementioned powdery clusters beneath the surface. Meanwhile, in the specimen subjected to the reaction at 280 °C, a similar occurrence of separation between the Ga_7_Ni_3_ and Ga-Cu IMC layers, as seen in Figure 4d, was witnessed in Figure 5d. However, in this instance, it is noticeable that the Ga_x_Ni phases emerge sporadically within a section of the region infiltrated by Ga. This is accompanied by a more pronounced separation between the IMC layers.

**Figure 6:**
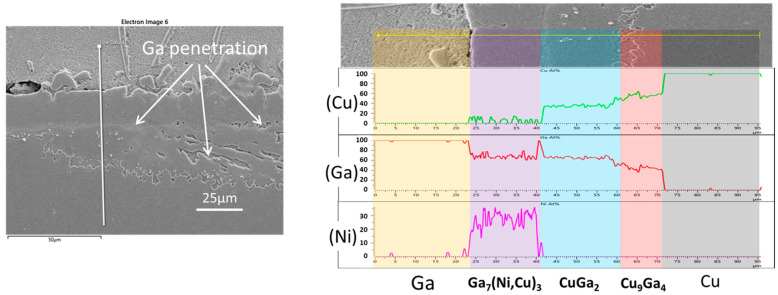
Compositional analysis using an EDX line scan of the Cu/Ni/Au-Ga specimen that reacted at 280 °C after 150 min.

An EDX line scan was conducted on the Cu/Ni/Au-Ga specimen that underwent extended reaction for 150 min at 280 °C to confirm the developed phases at the interface. The findings are depicted in Figure 6. In the upper section of the IMC layer, a substantial Ga_7_Ni_3_ layer is evident. A certain portion of Cu infiltrated Ni atomic sites, resulting in the creation of the Ga_7_(Ni,Cu)_3_ phase. The phenomenon of mutual substitution between Cu and Ni atoms in IMCs has been often found. For example, the literature has documented the formation of the (Ni,Cu)_3_Sn_4_ phase through Cu substitution into the Ni sites during reactions between Sn-based solders and Ni metallization [22,23,24]. Moving to the lower region, two distinct Ga-Cu IMCs are distinguishable, namely, the CuGa_2_ and Cu_9_Ga_4_ phases. However, the demarcation line between Cu_9_Ga_4_ and Cu is somewhat indistinct. Notably, Ga infiltration is also noticeable around the CuGa_2_ IMC layer. The observed microstructure aligns well with that of the Cu/Ni/Au-Ga specimen subjected to a 280 °C reaction for 210 min, presented in Figure 4d. These micrographs indicate that the extent of the separation between the Ga_7_Ni_3_ and Ga-Cu IMC layers becomes progressively more pronounced with increasing reaction time.

**Figure 7:**
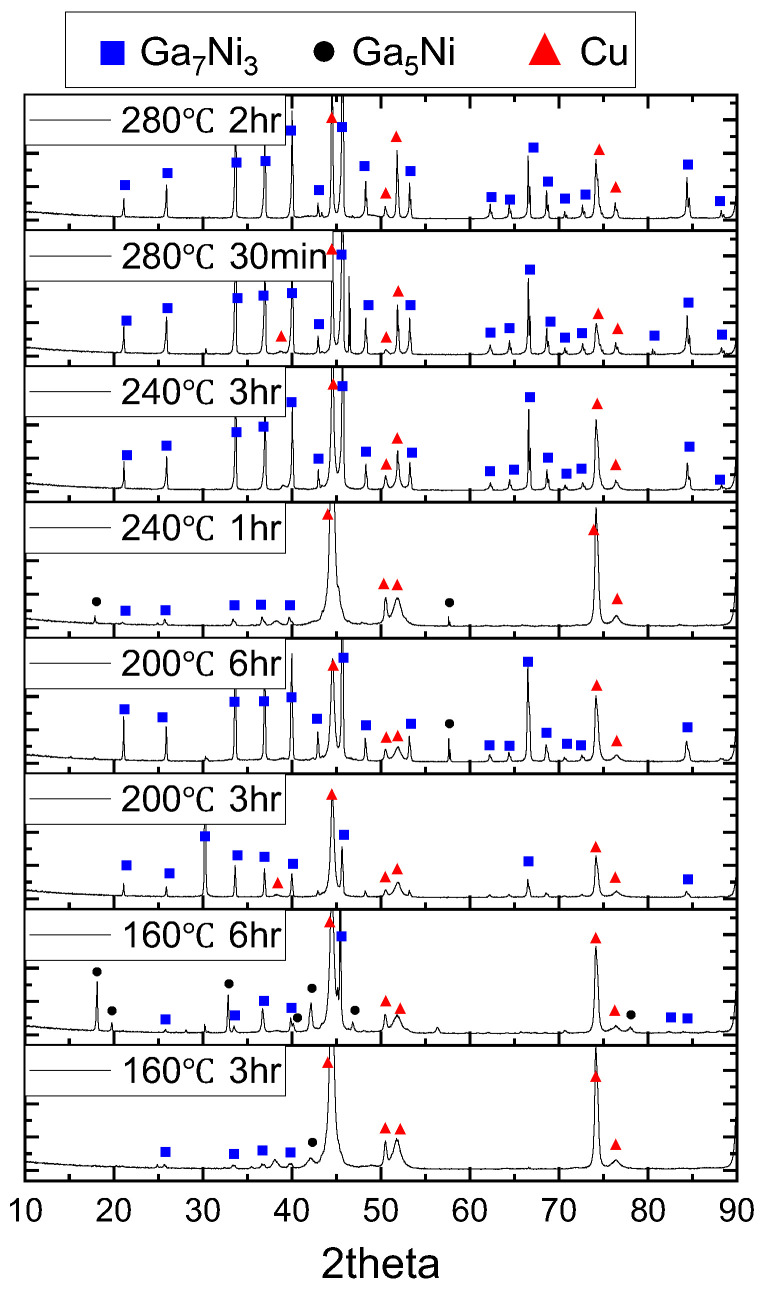
X-ray diffraction data for the IMCs that formed at Cu/Ni/Pd-Ga reaction interfaces. Each specimen was prepared after complete etching of unreacted Ga.

The specimens subjected to various reaction temperatures were applied to XRD analysis to ascertain the phases developed at the reaction interfaces. The residual unreacted Ga was effectively removed by overnight etching using the etching solution. The XRD patterns are presented in Figure 7. As a result of the analysis, three phases of Ga_7_Ni_3_ (ICSD ID 408313, space group Im-3m (229)), Ga_5_Ni (ICSD ID 165724, space group I4/mcm (140)), and Cu substrate were detected. Across all specimens, the dominant phase observed was Ga_7_Ni_3_. Prominent diffraction planes, namely, (013), (222), (123), (033), and (006), were discernible at diffraction angles of 2θ = 33.6, 36.9, 39.9, 45.6, and 66.5 degrees. The intensity of these diffraction peaks increased with elevated reaction temperature and extended reaction duration, attributable to the growth of the Ga_7_Ni_3_ phase during the Ga-Ni reactions. However, the Ga-rich Ga_x_Ni phase was not detected through XRD analysis. This phase is noted to possess a nonstoichiometric composition of Ga ranging from 89% to 95% at% [15].

There are two plausible explanations for the absence of the Ga_x_Ni phase in the XRD analysis. One possibility is that the Ga_x_Ni phase lacked chemical stability and was eliminated during the etching process applied to the specimens. Alternatively, the Ga_x_Ni phase could potentially exhibit a non-perfectly crystalline state, such as nano-crystalline or amorphous, rendering it less amenable to detection via XRD analysis. In either scenario, it is noteworthy that the Ga_x_Ni phase does not find representation in the Ga-Ni phase diagram [25,26], suggesting a high likelihood of its existence in a metastable state.

In the Ga-Ni binary phase diagram [26], the phase boasting the highest Ga content corresponds to Ga_5_Ni. During the XRD analysis, a discernible diffraction pattern was solely observed at 160 °C after a reaction time of 6 h. Upon examining the IMCs exposed on the surface after the complete etching of Ga, occasional instances of Ga_5_Ni IMC colonies came to light. These Ga_5_Ni IMC colonies present a distinctive rigid plate-like structure and showcase a balanced Ga:Ni stoichiometric ratio of 5:1. The distinctions between the two Ga-rich phases, namely, Ga_x_Ni and Ga_5_Ni, will be expounded upon in greater detail in the forthcoming chapter.

### 3.2. Kinetic Analysis of Ga_7_Ni_3_ IMC Growth

Throughout the reactions involving a Cu/Ni/Pd(Au)-Ga system, a range of distinct IMCs emerged, including Ga_x_Ni, Ga_5_Ni, Ga_7_Ni_3_, CuGa_2_, and Cu_9_Ga_4_. Notably, among these IMCs, the Ga_7_Ni_3_ phase exhibited persistent growth across all specimens during the course of the reactions. This observation prompted the present study to conduct a thorough kinetic analysis centered on tracking the growth of the Ga_7_Ni_3_ phase. To facilitate this analysis, a Cu/Ni/Pd structure was employed. This is because the Ni layer is thicker for the Cu/Ni/Pd structure and contributes to the growth of Ga_7_Ni_3_ IMC over a longer reaction time.

**Figure 8:**
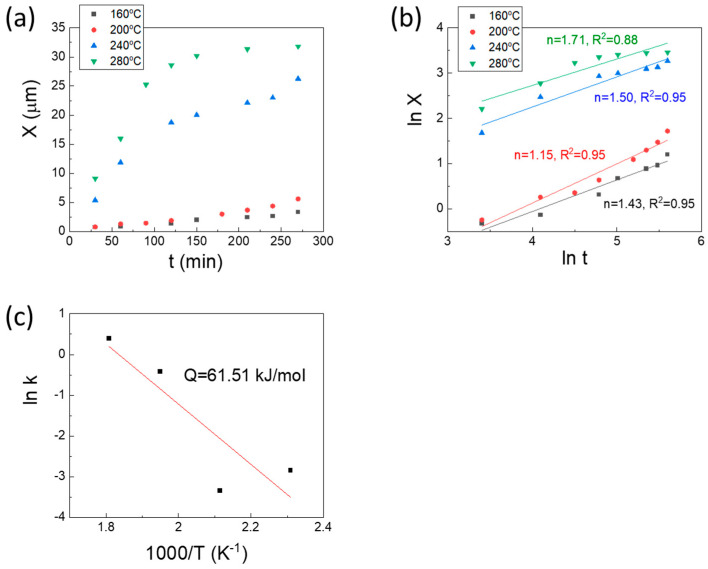
(**a**) Measured thickness of the Ga_7_Ni_3_ IMCs that formed at Cu/Ni/Pd-Ga reaction interfaces; (**b**) Log-log plot of Ga_7_Ni_3_ IMC growth rate with estimated time exponents; (**c**) Arrhenius plot for Ga_7_Ni_3_ IMC growth.

The variations in Ga_7_Ni_3_ IMC thickness concerning reaction temperature and time are illustrated in Figure 8a. Notably, a pronounced escalation in IMC growth rate becomes apparent beyond the 220 °C threshold. Within this realm of high-temperature reactions, a rapid proliferation of the Ga_7_Ni_3_ phase occurs for approximately 120 min, after which the growth rate experiences a notable decline. Figure 8b portrays a log-log plot detailing the correlation between interfacial Ga_7_Ni_3_ IMC thickness and reaction time. As is often the case, the empirical kinetic equation governing the IMC growth is formulated in the structure of Equation (1).

(1) $$X=kt^{1/n}=k_{0}exp(-\frac{Q}{RT})t^{1/n}$$

where X denotes the IMC thickness, t signifies the reaction time, T represents the temperature, and Q stands for the activation energy. Additionally, k, R, and n, respectively, denote the kinetic constant, gas constant, and time exponent. The calculated time exponents derived from the graph were 1.43, 1.15, 1.50, and 1.71 for temperatures of 160, 200, 240, and 280 °C, respectively.

Lee et al. [15] previously documented that the time exponents governing Ga_7_Ni_3_ growth ranged between 1.1 and 1.5 during reactions between Ga and Ni occurring at temperatures spanning from 250 to 350 °C. This behavior was understood to be underpinned by kinetics controlled by an interface reaction, facilitated by short-range diffusion. The limited diffusion range was attributed to the porous microstructure characterizing the Ga_7_Ni_3_ IMC layer. Consistently, within the scope of this investigation, a time exponent ranging approximately from 1.1 to 1.7 was derived. These outcomes underscore the substantial reliance of Ga_7_Ni_3_ IMC growth on interface reaction-controlled kinetics, even at low reaction temperatures spanning from 160 to 240 °C.

The determined activation energy for Ga_7_Ni_3_ growth in Cu/Ni/Pd-Ga reactions stands at 61.5 kJ/mol, slightly surpassing the 49.1 kJ/mol recorded for Ga-Ni reactions [15] yet remaining within a reasonable range. For the growth of the Ga_7_Ni_3_ compound, it becomes imperative to consider two distinct paths of atomic transport to establish the step that dictates the rate of growth. The first path entails the diffusion of Ni atoms into the molten Ga. Through tracer diffusion experiments, Eriksson et al. [27] determined Q for ^198^Au diffusion within liquid Ga, arriving at a value of 12.13 kJ/mol across the temperature range from 38 to 227 °C. This value is notably smaller than the measured Q of 61.5 kJ/mol for Ga_7_Ni_3_ growth within the 160–240 °C range.

The second pathway encompasses the diffusion of Ga atoms through the grain boundaries of Ga_7_Ni_3_ IMCs. Čermák et al. [28] reported a Q of 194.7 kJ/mol for Ni and Ga diffusion through Ni_3_Ga grain boundaries at temperatures of 700–1300 K. Stloukal et al. [29] reported a Q of 101 kJ/mol for grain boundary diffusion of ^67^Ga within polycrystalline magnesium, at a temperature range from 639 to 872 K. In a similar vein, Lohmann et al. [30] put forth a value of 54.8 kJ/mol for the solute diffusion of Ga within aluminum grain boundaries, at temperatures from 450 to 680 K. Building upon these findings, the diffusion of Ga atoms along grain boundaries is deemed a more plausible contender for the rate-determining step in Ga_7_Ni_3_ growth. Furthermore, when considering more networks of diffusion pathways due to the porous microstructure inherent in the Ga_7_Ni_3_ layer, it becomes evident that the activation energy could be further diminished for the reported values.

### 3.3. Morphological Characteristics of the Interfacial IMCs

In this study, throughout the reactions involving Cu/Ni/Au(Pd) and Ga at temperatures of 160–240 °C, a variety of IMCs are generated. Within these, the Ga-Ni IMCs encompass the Ga_x_Ni, Ga_5_Ni, and Ga_7_Ni_3_ phases, while the Ga-Cu IMCs consist of the CuGa_2_ and Cu_9_Ga_4_ phases. During the Ga-Ni reaction, two Ga-rich phases are generated: Ga_x_Ni (x = 89–95 at%) and Ga_5_Ni. The Ga_x_Ni phase is formed during the cooling process, not at the reaction temperature, and is deposited on top of the IMC layer. It is considered as a metastable phase [15]. This phase is formed in large quantities and is easily observed in a cross-sectional analysis, but it is easily lost during the process of etching all of the bulk Ga for the XRD analysis, making it difficult to observe in surface analysis. This absence may be attributed to its lack of chemical stability, which implies that it may have been eradicated during the Ga etching process or possibly dislodged from the surface during subsequent cleaning procedures.

**Figure 9:**
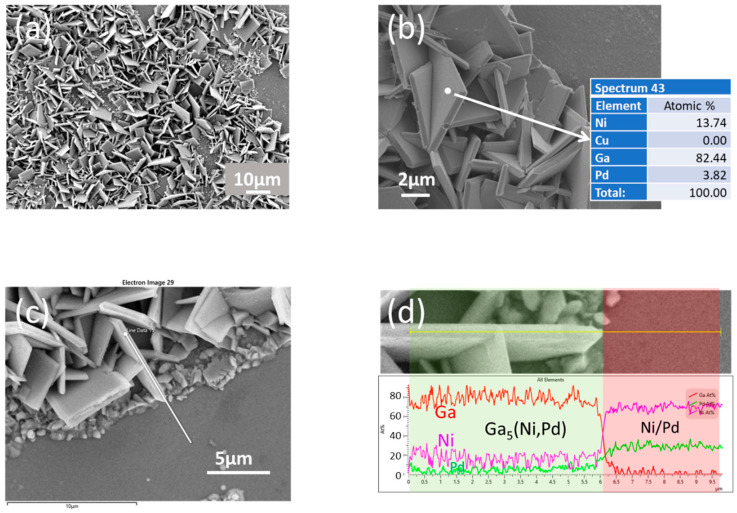
(**a**) Morphology of the Ga_5_Ni IMCs formed in the Cu/Ni/Pd-Ga specimen subjected to the reaction at 160 °C for 6 h, with EDX compositional analysis: (**b**) point scan and (**c**,**d**) line scan.

On the other hand, the Ga_5_Ni phase is observed in small amounts only in some specimens but has a stable composition and structure, so it can be observed on the surface even after bulk Ga etching. However, because the amount produced is small, it is not easy to find the part formed after the reaction. During the Cu/Ni/Pd-Ga reaction, the Ga_5_Ni diffraction pattern was substantiated in the sample subjected to 160 °C for 6 h. The distinctive morphology of the Ga_5_Ni IMC is depicted in Figure 9 for this particular specimen. Unlike the Ga_x_Ni phase, the Ga_5_Ni IMC showcases a robust plate-like structure and maintains a composition that remains stable stoichiometrically. This compositional stability was validated through EDX point and line scans, as shown in Figure 9b,d. From the analysis, it is assumed that a minor quantity of Pd exists within the compound in the configuration of Ga_5_(Ni,Pd), substituting the Ni position.

**Figure 10:**
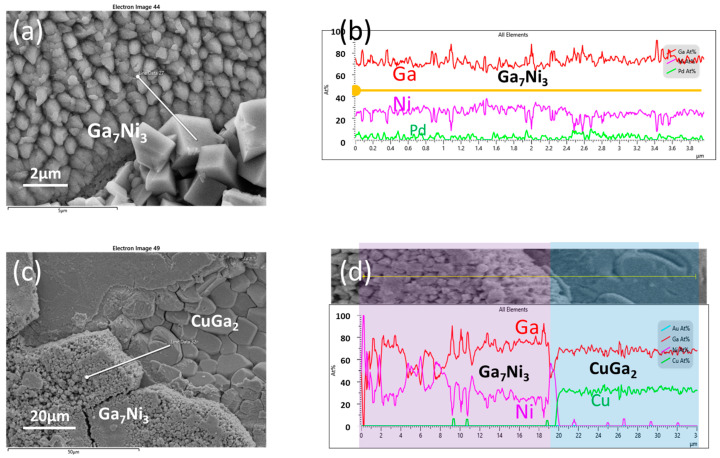
(**a**) Morphology of the Ga_7_Ni_3_ IMCs formed in the Cu/Ni/Pd-Ga specimen (6 h at 200 °C), (**b**) EDX line scan of (**a**), (**c**) morphology of thick Ga_7_Ni_3_ layer on the CuGa_2_ IMCs formed in the Cu/Ni/Au-Ga specimen (2 h at 280 °C), (**d**) EDX line scan of (**c**).

The Ga_7_Ni_3_ IMC grows as a single layer composed of numerous grains, yet a portion of them situated on top of the IMC layer, with an angular lump shape, is depicted in Figure 4c. Displayed in Figure 10a is the exposed surface of the Ga_7_Ni_3_ layer subsequent to thorough etching of the residual Ga, following the reaction of Cu/Ni/Pd and Ga at 200 °C for 6 h. In the lower right of the micrograph, there are some Ga_7_Ni_3_ lumps on the surface of the Ga_7_Ni_3_ lower layer, and it can be confirmed, with the EDX line scan result in Figure 10b, that they all have the same composition as Ga_7_Ni_3_.

With the progressive increase in reaction temperature or reaction duration, a transformation takes place wherein the Ni layer is gradually consumed and replaced by Ga-Ni IMCs. This change facilitates the interdiffusion of Ga and Cu through grain boundaries of the Ga-Ni IMCs. In response, the underlying Cu layer undergoes a reaction, culminating in the formation of Ga-Cu IMCs, specifically CuGa_2_ and Cu_9_Ga_4_, as visualized in Figure 4d and Figure 5d. Figure 10c offers a portrayal of the interface established after the reaction between Cu/Ni/Au and Ga at 280 °C for 2 h. Notably, it becomes evident that the CuGa_2_ IMC layer takes shape beneath the Ga_7_Ni_3_ layer. As previously, the elemental composition of each layer is corroborated via EDX line scans. In the microstructure, it is noteworthy that the grain size of CuGa_2_ surpasses that of Ga_7_Ni_3_ by an order of magnitude. Meanwhile, during the reactions, Cu atoms undergo diffusion into the molten Ga, leading to the creation of considerably large plate-like CuGa_2_ IMCs within the bulk Ga. Some of these IMCs attain sizes reaching several hundred micrometers (see Supplementary Materials).

## 4. Conclusions

In this study, practical metallization of Cu/Ni/Pd and Cu/Ni/Au applied to modern microelectronic packages are introduced to simulate low-temperature soldering with Ga. With these multilayer structures, the stability of the Ni diffusion barrier was investigated by observing the change of interfacial microstructure according to Ni consumption. Useful data for low-temperature soldering with Ga are drawn and the conclusions can be summarized as follows.

(1) When the reaction temperature is low, the presence of Ga-rich Ga_x_Ni (x = 89–95 at%) IMCs on top of the Ga_7_Ni_3_ layer was distinctly verified. As the reaction temperature and duration increase, the Ni layer becomes progressively consumed. This results in the formation of Ga-Cu IMCs, specifically CuGa_2_ and γ3-Cu_9_Ga_4_, beneath the Ga-Ni IMC layer. Simultaneously, the gap between the Ga-Ni and Ga-Cu IMC layers widens, creating space for molten Ga to infiltrate.
(2) The time exponent for Ga_7_Ni_3_ growth was determined to range approximately from 1.1 to 1.7. These outcomes underscore the substantial reliance of Ga_7_Ni_3_ IMC growth on interface reaction-controlled kinetics, at low reaction temperatures spanning from 160 to 240 °C.
(3) The determined activation energy for Ga_7_Ni_3_ growth in Cu/Ni/Pd-Ga reactions stands at 61.5 kJ/mol. The diffusion of Ga atoms along grain boundaries, with more diffusion pathways due to the porous microstructure inherent in the Ga_7_Ni_3_ layer, is assumed for the rate-controlling step in Ga_7_Ni_3_ growth.
(4) There are two types of Ga-rich IMCs formed: Ga_5_Ni and Ga_x_Ni. The Ga_x_Ni is assumed to be a metastable phase, not accounted for in the phase diagram. This phase was not identified in the XRD analysis, likely due to chemical instability or insufficient crystallinity. On the other hand, the Ga_5_Ni phase, which possesses a stable, plate-like structure, maintains a consistent composition with stoichiometric uniformity.

## Supplementary Materials

The following supporting information can be downloaded at: https://www.mdpi.com/article/10.3390/ma16186186/s1, Figure S1: Crystallographic information of the Cu_9_Ga_4_ phase and large CuGa_2_ plates formed in the Cu/Ni/Au-Ga specimen.
