# Supplementary material for: A Study on the Interfacial Reactions between Gallium and Cu/Ni/Au(Pd) Multilayer Metallization
Source: Materials (Basel). 2023 Sep 13;16(18):6186. doi: 10.3390/ma16186186 (PMC10532968; doi:10.3390/ma16186186)
Supplement: Supplementary file 1 [file materials-16-06186-s001.zip › materials-2597811-supplementary.pdf]

materials

Supplementary Materials

A Study on the Interfacial Reactions between Gallium and Cu/Ni/Au(Pd) Multilayer Metallization

Byungwoo Kim [1,2,†], Chang-Lae Kim [3,†] and Yoonchul Sohn [1,*]

[1] Department of Welding and Joining Science Engineering, Chosun University, Gwangju 61452, Republic of Korea
[2] Solder R&D Team, MK Electron Co., Ltd., Yongin 449-812, Republic of Korea
[3] Department of Mechanical Engineering, Chosun University, Gwangju 61452, Republic of Korea
* Correspondence: yoonchul.son@chosun.ac.kr
† These authors contributed equally to this work.

Crystallographic information of the Cu_9Ga_4 phase (**JCPDS** No. 02-1253)
(copy from Springer Materials homepage: https://materials.springer.com/isp/crystallographic/docs/sd_0526925)

General Information

Phase Label(s): Cu_9Ga_4 rt
Structure Class(es): γ-brass
Classification by Properties: –
Mineral Name(s): –
Pearson Symbol: *cP*52
Space Group: 215
Phase Prototype: Cu_9Al_4
Measurement Detail(s): film (determination of cell parameters), X-rays, Cu Kα (determination of cell parameters)
Phase Class(es): –
Compound Class(es): intermetallic
Interpretation Detail(s): cell parameters determined and structure type assigned; composition dependence studied
Sample Detail(s): powder (determination of cell parameters)

Large $CuGa_2$ plates formed in the Cu/Ni/Au-Ga specimen subjected to the reaction at 280 °C for 150 min

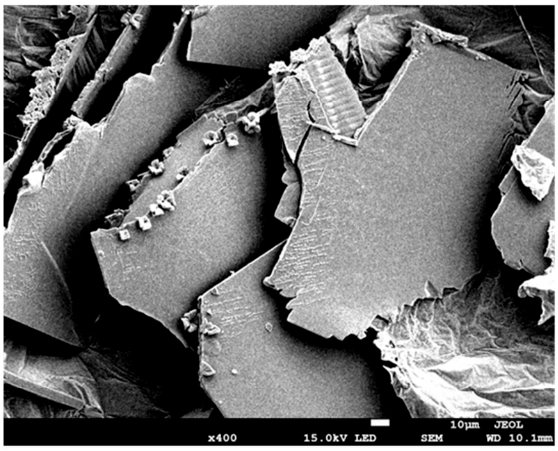

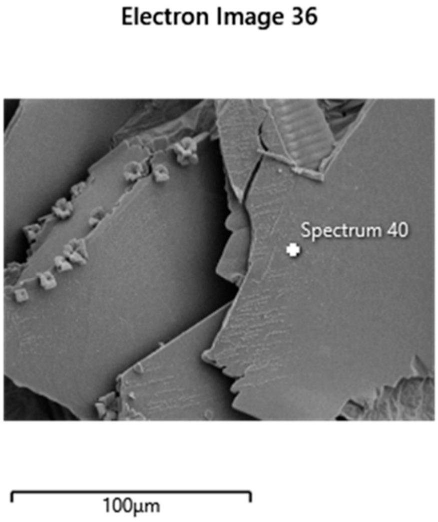

Spectrum 40	
Element	Atomic %
Ni	0.50
Cu	33.00
Ga	66.49
Au	0.00
Total:	100.00

Figure S1. Crystallographic information of the Cu_9Ga_4 phase and large $CuGa_2$ plates formed in the Cu/Ni/Au-Ga specimen.
